# Large Cell Neuroendocrine Carcinoma of the Bladder with Adenocarcinomatous Component

**DOI:** 10.1155/2020/8827646

**Published:** 2020-10-05

**Authors:** Rami Halabi, Maher Abdessater, Johnny Boustany, Anthony Kanbar, Halim Akl, Joey El Khoury, Charbel El Hachem, Raghid El Khoury

**Affiliations:** ^1^Department of Urology, Notre Dame des Secours University Hospital Center (CHUNDS), Byblos City, Lebanon; ^2^Faculty of Medicine and Medical Sciences, Holy Spirit University of Kaslik (USEK), Jounieh, Lebanon

## Abstract

Large cell neuroendocrine carcinoma (LCNC) is one of the rarest types of bladder cancer occurring in <1%. Either pure or mixed with another component, it remains one of the most aggressive types of bladder cancer. We report a case of LCNC of the bladder with an adenocarcinomatous component. The patient was a 64-year-old smoker male, who presented for the first time with dysuria and hematuria. A bladder tumor invading the anterior and right lateral bladder walls was discovered, without any secondary localizations. Tumor biopsy showed an LCNC with adenocarcinomatous components. The patient was treated by recurrent tumor resections, chemotherapy, and radiotherapy. No improvement was noted despite close follow-up and adequate treatment. Neuroendocrine bladder tumor is known to have an aggressive, rapid, and disadvantageous evolution. Multiple case reports were published so far, and a recent review was conducted in March 2020 by Sanguedolce et al. (2020). More cases are needed to establish the best management plan for this type of tumor.

## 1. Introduction

Bladder cancer (BC) is the 6th most frequent neoplasm in men and the 17th most frequent in women [1]. Its annual incidence increased from 430000 cases in 2012 to 550000 in 2018 [1, 2]. Lebanon was found to have the world's highest rate of BC [1]. According to the World Health Organization classification, more than 90% of BC are urothelial and around 5% are squamous cell carcinoma. The neuroendocrine bladder carcinoma is considered a rare variant (<1%) with highly aggressive potential. These tumors are divided into small cell neuroendocrine carcinoma, large cell neuroendocrine carcinoma, well-differentiated neuroendocrine tumor, and paraganglioma [2]. The small cell type is the most common, and the large cell neuroendocrine cancer type (LCNC) is the rarest. Less than forty cases of LCNC were reported so far in the literature. Only three cases had histological similarities to our case [3–5]. Management strategies are not yet studied prospectively [6, 7]. In this article, we report a case of LCNC with an adenocarcinomatous component.

## 2. Case Presentation

**Figure 1:**
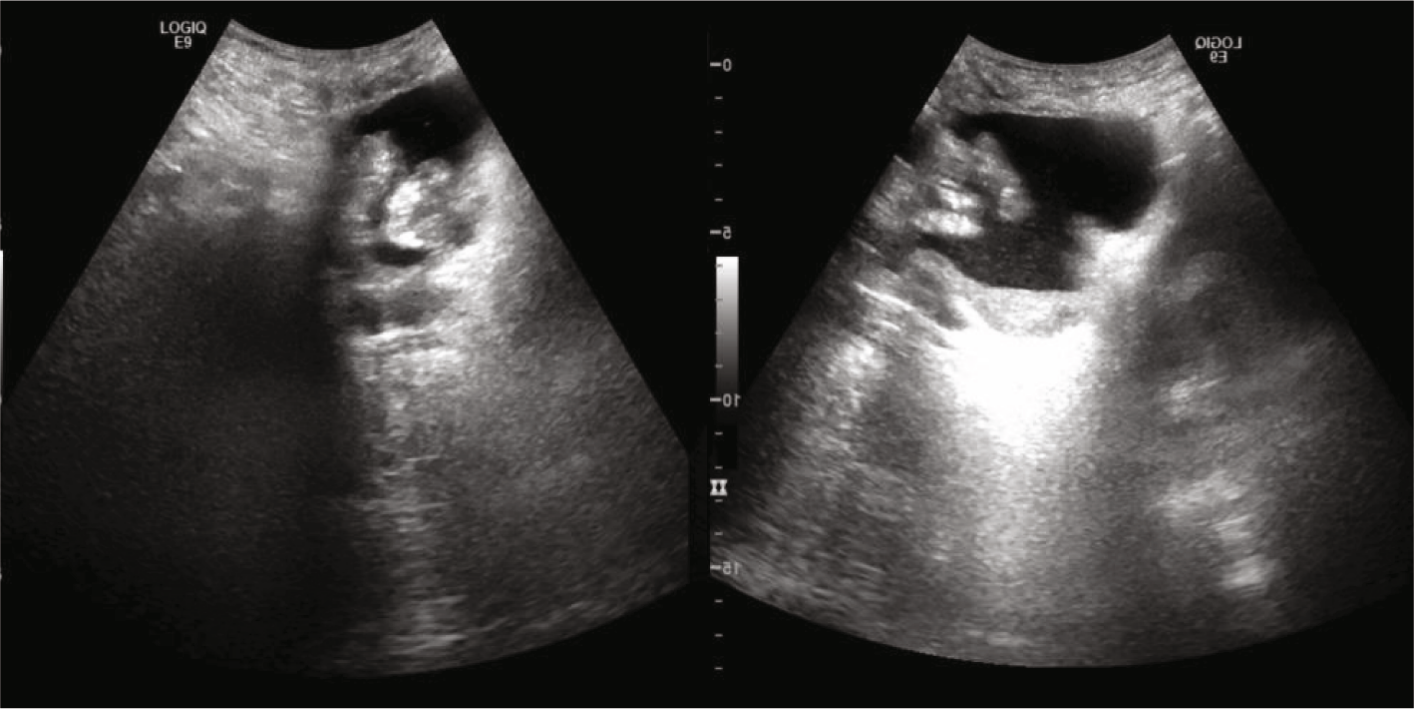
The first bladder echography showing a thickening of the right lateral wall.

**Figure 2:**
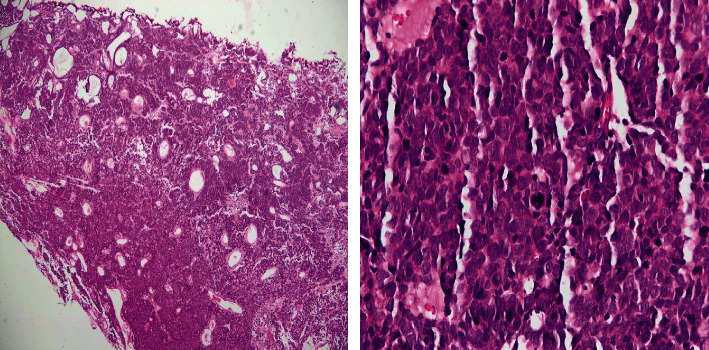
On the left: microscopic view of the bladder biopsy showing a proliferation of polygonal malignant cells and cylindrical cells with multiple necrotic areas; on the right: large polygonal cells with a dark and irregular nucleus and frequent metastases.

**Figure 3:**
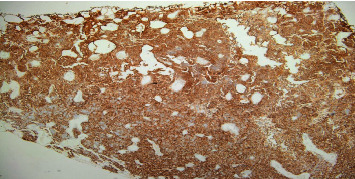
Malignant cells showing high positivity for CD56 marker on immunohistochemistry.

**Figure 4:**
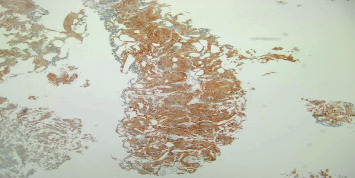
Malignant cells showing high positivity of synaptophysin marker on immunohistochemistry.

**Figure 5:**
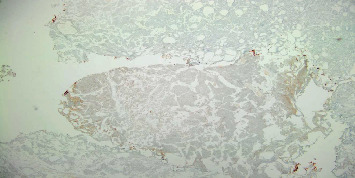
Malignant cells showing minor and heterogenic positivity for cytokeratin 7 marker on immunohistochemistry.

**Figure 6:**
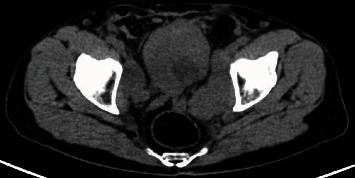
Pelvic scan showing a 6 × 3.5 cm mass of the anterior and right lateral wall of the bladder with diffuse infiltration of the bladder fat.

**Figure 7:**
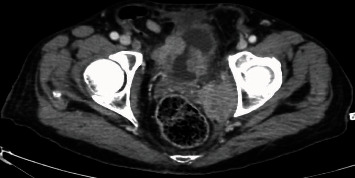
Pelvic scan showing the evolution of the bladder tumor.

A 64-year-old heavy smoker male patient was admitted for the first time to our urology department for hematuria and dysuria. His past medical history was unremarkable. He had a transurethral resection of the prostate 5 years ago. He did not have any familial history of urothelial or bladder cancer. Physical examination and blood tests were normal. In the emergency room, an abdominal and pelvic ultrasound showed a thickening of the right lateral wall of the bladder with mild right side hydronephrosis (Figure 1). On cystoscopy, a large tumor was found on the anterior and right lateral walls of the bladder, with invasion of the bladder neck and the prostate. The anatomopathological study of the tumor showed a neoplastic proliferation of large malignant polygonal cells with dark, irregular nuclei and frequent mitosis. A second component made of cylindrical cells was also present. Muscular invasion was documented with multiple necrotic areas (Figure 2). Immunohistochemistry showed high positivity for CD56 marker and synaptophysin, minor and heterogenic positivity for cytokeratin 7, and negativity for cytokeratin 20 and CDX2 (Figures 3–5). Overall, the histopathological study corresponded to LCNC with minor adenocarcinomatous component. Disease extension was assessed using computed tomography (CT) scan of the thorax, abdomen, and pelvis. A 6 × 3.5 cm mass of the anterior and right lateral walls of the bladder was identified, with diffuse infiltration of the perivesical fat and multiple necrotic iliac lymph nodes bilaterally (Figure 6). Neither visceral nor bone metastasis was identified. Initially, the patient refused the treatment and was lost to follow-up. Two months later, he was readmitted to the hospital for similar complaints and additional pelvic pain. Blood tests were normal. A new CT scan revealed an increase in the size of the tumor, occupying now the majority of the bladder lumen, a persistent right hydronephrosis, and no new secondary lesions. The tumor was resected again, until the appearance of the muscular layer of the bladder, and the patient was discharged after stabilization. A later PET CT confirmed the previous findings of locoregional disease (tumor of the bladder base with infiltration of the prostate gland and a diffuse metastatic bilateral iliac chain and retroperitoneal lymph nodes) and denied secondary lesions. The multidisciplinary team meeting decided for radiotherapy and chemotherapy using cisplatin and etoposide every 21 days. Four months later, after the completion of four cycles of chemotherapy, the patient underwent new assessment for disease extension (Figure 7). CT scan showed progression of the disease with the tumor occupying now the totality of the bladder lumen, infiltration of the anterior abdominal wall, and subcutaneous liquid collection measuring 33 × 36 mm. A similar left retrovesical mass of 60 × 38 mm along with multiple para-aortic and mediastinal lymphadenopathies with central necrosis was also found. A few months later, the patient died in the oncology department after recurrent admissions for different complications.

## 3. Discussion

Neuroendocrine carcinomas are most frequently seen in the pulmonary and gastrointestinal tracts. This type of tumor can be also found in the urinary tract. According to the literature, different types of neuroendocrine carcinomas of the bladder were described. The LCNC is the rarest type [8, 9]. It can present as a pure tumor, or in association with carcinosarcoma, adenocarcinoma or lymphoepithelioma-like carcinoma.

The first published case by Abenoza et al. had mixed histological features of LCNC and adenocarcinomatous cells. It was treated by a radical cystectomy and bilateral lymphadenectomy. The patient died 30 months after the surgery [9]. Subsequently, less than forty cases of pure and mixed LCNC were published [3, 4, 6–8, 10–21]. To our knowledge, only three cases showed similar histological characteristics to our case, with LCNC and adenocarcinoma components [7, 17, 22].

Only two of the published cases of LCNC were discovered from cutaneous or a brain metastasis [10, 13]. Our patient presented local infiltration of the prostatic gland with bilateral iliac and retroperitoneal lymph nodes. Although the latter was a frequent finding in the literature, the former has never been described [23].

The clinical presentation of our patient was a typical presentation of bladder cancer, similar to the previously published cases, in which hematuria was a frequent presentation [3–5, 7, 8, 12, 13, 17–19].

No guidelines were ever published concerning the treatment modalities of LCNC. A cisplatin-based chemotherapy consisted the base of the treatment strategies through literature. A multimodal therapeutic approach, using the combination of chemotherapy, radiotherapy, and radical or partial surgeries, has been reported [3, 4, 25, 27]. Dowd et al. reported after the treatment by surgical resection and adjuvant chemotherapy and radiotherapy, no recurrence at one year [4]. In their study in 2018 on thirty-five cases of LCNC, Niu et al. reported higher survival rates in patients treated by multimodal therapies compared to patients treated with a conservative treatment. They reported also that a platine-based chemotherapy was the most used option [28]. The management plan of our patient was similar to Dowd et al.'s plan. Our patient was managed by repetitive transurethral tumor resection of the bladder tumor, multiple cycles of cisplatin and etoposide chemotherapy, and radiotherapy.

Coelho et al. have stated in their case report and review that LCNC has a bad prognosis and is usually discovered in an advanced or metastatic stage [7]. A bibliographic review in 2011 by Martin et al. showed that the survival rate for all published cases concerning LCNC was 46.2% for a maximum follow-up of 20 months [23], which was the case of our patient.

## 4. Conclusion

Bladder tumor incidence is increasing nowadays. Neuroendocrine type is a rare one, with aggressive, rapid, and disadvantageous evolution. So far, we lack prospective studies concerning therapeutic strategies, and management is based on some previous published case reports.

## References

[1] Ferlay J., Colombet M., Soerjomataram I. (2019). Estimating the global cancer incidence and mortality in 2018: GLOBOCAN sources and methods. *International Journal of Cancer*.

[2] Ferlay J., Soerjomataram I., Dikshit R. (2015). Cancer incidence and mortality worldwide: sources, methods and major patterns in GLOBOCAN 2012. *International Journal of Cancer*.

[3] Akdeniz E., Bakirtas M., Bolat M. S., Akdeniz S., Özer I. (2018). Pure large cell neuroendocrine carcinoma of the bladder without urological symptoms. *Pan African Medical Journal*.

[4] Dowd K., Rotenberry C., Russell D., Wachtel M., De Riese W. (2017). Rare occurrence of a poorly differentiated neuroendocrine tumor of the bladder. *Case Reports in Medicine*.

[5] Thota S., Kistangari G., Daw H., Spiro T. (2013). A clinical review of small-cell carcinoma of the urinary bladder. *Clinical Genitourinary Cancer*.

[6] Bertaccini A., Marchiori D., Cricca A. (2008). Neuroendocrine carcinoma of the urinary bladder: case report and review of the literature. *Anticancer Research*.

[7] Coelho H. M. P., Pereira B. A. G. J., Temido P. A. S. C. (2013). Large cell neuroendocrine carcinoma of the urinary bladder: case report and review. *Current Urology*.

[8] Lee K. H., Ryu S. B., Lee M. C., Park C. S., Juhng S. W., Choi C. (2006). Primary large cell neuroendocrine carcinoma of the urinary bladder. *Pathology International*.

[9] Mills S. E., Wolfe J. T., Weiss M. A. (1987). Small cell undifferentiated carcinoma of the urinary bladder. A light-microscopic, immunocytochemical, and ultrastructural study of 12 cases. *The American Journal of Surgical Pathology*.

[10] Lee W. J., Kim C. H., Chang S. E. (2009). Cutaneous metastasis from large-cell neuroendocrine carcinoma of the urinary bladder expressing CK20 and TTF-1. *The American Journal of Dermatopathology*.

[11] Wang J. (2014). Large cell neuroendocrine carcinoma of the urinary bladder: a pooled analysis. *Journal of Clinical Oncology*.

[12] Oshiro H., Gomi K., Nagahama K. (2010). Urinary cytologic features of primary large cell neuroendocrine carcinoma of the urinary bladder: a case report. *Acta Cytologica*.

[13] Tsugu A., Yoshiyama M., Matsumae M. (2011). Brain metastasis from large cell neuroendocrine carcinoma of the urinary bladder. *Surgical Neurology International*.

[14] Sroussi M., Lorcet M., Tardy M. P. (2018). Neuroendocrine carcinoma of the urinary bladder: a large analysis of the French GETUG consortium. *Annals of Oncology*.

[15] Dundr P., Pesl M., Povysil C., Vitkova I., Dvoracek J. (2003). Large cell neuroendocrine carcinoma of the urinary bladder with lymphoepithelioma-like features. *Pathology, Research and Practice*.

[16] Li Y., Outman J. E., Mathur S. C. (2004). Carcinosarcoma with a large cell neuroendocrine epithelial component: first report of an unusual biphasic tumour of the urinary bladder. *Journal of Clinical Pathology*.

[17] Evans A. J., Al-Maghrabi J., Tsihlias J., Lajoie G., Sweet J. M., Chapman W. B. (2002). Primary large cell neuroendocrine carcinoma of the urinary bladder. *Archives of Pathology & Laboratory Medicine*.

[18] Hailemariam S., Gaspert A., Komminoth P., Tamboli P., Amin M. (1998). Primary, pure, large-cell neuroendocrine carcinoma of the urinary bladder. *Modern Pathology*.

[19] Alijo Serrano F., Sanchez-Mora N., Angel Arranz J., Hernandez C., Alvarez-Fernandez E. (2007). Large cell and small cell neuroendocrine bladder carcinoma. *American Journal of Clinical Pathology*.

[20] Akamatsu S., Kanamaru S., Ishihara M., Sano T., Soeda A., Hashimoto K. (2008). Primary large cell neuroendocrine carcinoma of the urinary bladder. *International Journal of Urology*.

[21] Chong V., Zwi J., Hanning F., Lim R., Williams A. (2017). A case of large cell neuroendocrine carcinoma of the bladder with prolonged spontaneous remission. *Journal of Surgical Case Reports*.

[22] Abenoza P., Manivel C., Sibley R. K. (1986). Adenocarcinoma with neuroendocrine differentiation of the urinary bladder. Clinicopathologic, immunohistochemical, and ultrastructural study. *Archives of Pathology & Laboratory Medicine*.

[23] Martin I. J., Vilar D. G., Aguado J. M. (2011). Large cell neuroendocrine carcinoma of the urinary bladder. Bibliographic review. *Archivos Españoles de Urología*.

[24] Ghervan L., Zaharie A., Ene B., Elec F. I. (2017). Small-cell carcinoma of the urinary bladder: where do we stand?. *Medicine and Pharmacy Reports*.

[25] Quek M. L., Nichols P. W., Yamzon J. (2005). Radical cystectomy for primary neuroendocrine tumors of the bladder: the university of southern California experience. *The Journal of Urology*.

[26] Niu Q., Lu Y., Xu S. (2018). Clinicopathological characteristics and survival outcomes of bladder neuroendocrine carcinomas : a population-based study. *Cancer Management and Research*.

[27] Sanguedolce F., Calò B., Chirico M., Tortorella S., Carrieri G., Cormio L. (2020). Urinary tract large cell neuroendocrine carcinoma: diagnostic, prognostic and therapeutic issues. *Anticancer Research*.

[28] Moch H., Humphrey P. A., Ulbright T. M., Reuter V. E. (2016). WHO classification of tumours of the urinary system and male genital organs. *World Heal Organ Classifcation Tumours*.

